# Hepatic steatosis with significant fibrosis is associated with an increased 10-year estimated risk of cardiovascular disease in adults with type 1 diabetes mellitus

**DOI:** 10.1186/s12933-023-01945-x

**Published:** 2023-08-10

**Authors:** Alessandro Mantovani, Mario Luca Morieri, Luisa Palmisano, Maria Masulli, Efisio Cossu, Marco Giorgio Baroni, Katia Bonomo, Flavia Agata Cimini, Gisella Cavallo, Raffaella Buzzetti, Carmen Mignogna, Frida Leonetti, Simonetta Bacci, Roberto Trevisan, Riccardo Maria Pollis, Raffaella Aldigeri, Alessandra Dei Cas, Saula Vigili de Kreutzenberg, Giovanni Targher

**Affiliations:** 1 Section of Endocrinology, Diabetes and Metabolism, Department of Medicine, University of Verona, Verona, Italy; 2 Metabolic Diseases, Department of Medicine, University of Padua, Padua, Italy; 3 Department of Clinical Medicine and Surgery, Federico II University, Naples, Italy; 4 Diabetology Unit, Policlinico Universitario of Cagliari, Cagliari, Italy; 5 Department of Clinical Medicine, Life, Health and Environmental Sciences, University of Aquila, L’Aquila, Italy; 6 Neuroendocrinology and Metabolic Diseases, IRCCS Neuromed, Pozzilli, Italy; 7 Diabetes and Metabolic Diseases Unit, San Luigi Gonzaga University Hospital, Turin, Italy; 8 Department of Experimental Medicine, Sapienza University, Rome, Italy; 9 Department of Medical-Surgical Sciences and Biotechnologies, Sapienza University, Rome, Italy; 10 Section of Endocrinology, Department of Medicine, IRCCS Casa Sollievo della Sofferenza Hospital, San Giovanni Rotondo, Italy; 11 Department of Medicine and Surgery, University of Milan Bicocca, Milan, Italy; 12 Division of Nutritional and Metabolic Sciences, Azienda Ospedaliero-Universitaria, Parma, Italy; 13 Department of Medicine and Surgery, University of Parma, Parma, Italy; 14 IRCCS Sacro Cuore Don Calabria Hospital, Negrar di Valpolicella, VR Italy; 15 Section of Endocrinology, Diabetes and Metabolism, Department of Medicine, University and Azienda Ospedaliera Universitaria Integrata, Piazzale Stefani, 1, Verona, 37126 Italy

**Keywords:** NAFLD, Non-alcoholic fatty liver disease, T1DM, Type 1 diabetes, CVD, Cardiovascular disease

## Abstract

**Background:**

We assessed whether hepatic steatosis with or without significant fibrosis (determined by validated non-invasive biomarkers) is associated with an increased 10-year estimated risk for cardiovascular disease (CVD) in people with type 1 diabetes mellitus (T1DM).

**Methods:**

We conducted a retrospective, multicenter, cross-sectional study involving 1,254 adults with established T1DM without pre-existing CVD. We used the hepatic steatosis index (HSI) and fibrosis (FIB)-4 index for non-invasively detecting hepatic steatosis (defined as HSI > 36), with or without coexisting significant fibrosis (defined as FIB-4 index ≥ 1.3 or < 1.3). We calculated the Steno type 1 risk engine and the atherosclerotic CVD (ASCVD) risk score to estimate the 10-year risk of developing a first fatal or nonfatal CVD event.

**Results:**

Using the Steno type 1 risk engine, a significantly greater proportion of patients with hepatic steatosis and significant fibrosis (n = 91) had a high 10-year estimated CVD risk compared to those with hepatic steatosis alone (n = 509) or without steatosis (n = 654) (75.8% vs. 23.2% vs. 24.9%, p < 0.001). After adjustment for sex, BMI, diabetes duration, hemoglobin A1c, chronic kidney disease, and lipid-lowering medication use, patients with hepatic steatosis and significant fibrosis had an increased 10-year estimated risk of developing a first fatal or nonfatal CVD event (adjusted-odds ratio 11.4, 95% confidence interval 3.54–36.9) than those without steatosis. We observed almost identical results using the ASCVD risk calculator.

**Conclusions:**

The 10-year estimated CVD risk is remarkably greater in T1DM adults with hepatic steatosis and significant fibrosis than in their counterparts with hepatic steatosis alone or without steatosis.

**Supplementary Information:**

The online version contains supplementary material available at 10.1186/s12933-023-01945-x.

## Introduction

Non-alcoholic fatty liver disease (NAFLD) has become a widespread and fast-growing public health threat, causing substantial social and economic costs and reduced health-related quality of life [1, 2]. NAFLD affects almost a third of the world’s adult population [3], up to ~ 70% of individuals with type 2 diabetes mellitus (T2DM) [4], and up to ~ 40% of adults with type 1 diabetes mellitus (T1DM) [5]. NAFLD is a systemic disorder [6], which is associated not only with liver-related morbidity and mortality [7] but also with an increased risk of developing important extrahepatic complications [8], such as fatal and nonfatal cardiovascular disease (CVD) events and new-onset heart failure (HF) [9, 10]. Using the Korean National Health Insurance dataset, Park et al. recently reported that hepatic steatosis and/or advanced fibrosis as non-invasively assessed by fatty liver index and BARD score was significantly associated with an increased risk of HF and mortality [11] as well as with an increased risk of CVD events and mortality in new-onset T2DM [12]. Moreover, improvement in hepatic steatosis (assessed by changes in fatty liver index or BARD score) was significantly associated with decreased risk for HF and liver-related mortality as well as with decreased risk of CVD outcomes and mortality in new-onset T2DM [13, 14]. Strong evidence indicates that CVD is the leading cause of mortality in people with NAFLD, followed by extrahepatic malignancies and liver-related complications [15, 16].

CVD also represents the predominant cause of morbidity and mortality in adults with T1DM [17, 18], with an estimated loss of life expectancy at age 20 years of approximately 11 years for men and 13 years for women compared with the general population without T1DM [19]. Although NAFLD is associated with a substantial economic and health burden in individuals with T2DM, especially in terms of CVD mortality and morbidity [8], little information is currently available about the adverse effect of NAFLD on CVD risk in people with T1DM. Only a few small single-center studies examined the association of NAFLD with markers of subclinical atherosclerosis [20] or the risk of CVD outcomes in adults with T1DM [21–23].

Using quantitative risk assessment tools for estimating the risk of developing a first ‘hard’ CVD event in individuals with T1DM and NAFLD is an important starting point for clinicians to guide decision-making in the primary prevention of CVD. Many CVD risk scores have been proposed to estimate the 10-year risk for a first fatal and nonfatal CVD event, including the Steno type 1 risk engine [24], i.e., a CVD prediction calculator that is specific for adults with T1DM, and the atherosclerotic cardiovascular disease (ASCVD) risk calculator [25] that can be used for both adults with and without diabetes.

Thus, in this multicenter cross-sectional study, we aimed to explore whether hepatic steatosis (NAFLD) with and without significant fibrosis (as determined by validated non-invasive biomarkers) was associated with an increased 10-year estimated CVD risk in a large cohort of adults with T1DM without pre-existing CVD.

## Methods

### Participants

We performed a retrospective, multicenter, cross-sectional study on 11 Italian diabetes primary care outpatient clinics, all participating sites in the Study Group on Diabetes and Atherosclerosis of the Italian Society of Diabetes. More details about the recruitment methods of the study have been described elsewhere [26]. Briefly, all data were retrospectively retrieved from electronic medical records and patients’ medical charts in each participating center during 2018 and 2019 [26]. The inclusion criteria of the study were adult (age ≥ 18 years) individuals with established T1DM, according to the American Diabetes Association criteria [27]. Participants with T2DM or other specific types of diabetes, active cancer, and a history of chronic viral hepatitis or cirrhosis of any etiology were excluded. Participants with pre-existing ischemic heart disease, stroke, coronary or peripheral revascularization procedures, or missing data on platelet count, serum aminotransferase concentrations, and other variables that were used for calculating CVD risk scores and non-invasive biomarkers of hepatic steatosis and fibrosis (as described below), were also excluded. After excluding participants who did not meet inclusion criteria, the final sample for analysis consisted of 1,254 adult individuals (691 men and 563 women) with established T1DM (Supplementary Fig. 1). The study protocol was approved by the “Comitato etico per la Sperimentazione Clinica della Provincia di Padova” (code #63,553, October 2018) and by the ethics committee of each participating center. Written informed consent was collected accordingly to the requests of each local ethics committee [26].

### Clinical and laboratory data

Extracted electronic data were sex, age, diabetes duration, body mass index (BMI), blood pressure, and biochemical parameters, such as complete blood count, plasma lipids, glucose, hemoglobin A1c (HbA1c), creatinine and liver enzymes (aspartate aminotransferase [AST], alanine aminotransferase [ALT] and gamma-glutamyl transferase [GGT]). The estimated glomerular filtration rate (e-GFR) was calculated using the CKD Epidemiology Collaboration (CKD-EPI) equation. [28]. Abnormal albuminuria was defined as urine albumin-to-creatinine ratio (ACR) ≥ 3.0 mg/mmol. Smoking history was dichotomized as current (yes) or no smoker (no or former > 1 year) and regular physical exercise using a cut-point of > 3.5 h/week [26]. Significant alcohol intake was defined as ≥ 2 alcohol units per day in men and ≥ 1 alcohol unit per day in women, respectively. In all participants, information was also recorded on chronic kidney disease (CKD) (defined as e-GFR < 60 mL/min/1.73 m^2^ or urine ACR ≥ 3.0 mg/mmol), diabetic retinopathy of any degree, as well as total daily insulin doses and use of other concomitant drug treatments (including antihypertensive, antiplatelet or lipid-lowering medications) [26].

### Non-invasive biomarkers of hepatic steatosis and fibrosis

The hepatic steatosis index (HSI) was used to identify individuals with a high probability of hepatic steatosis. HSI was calculated as follows: HSI = 8 × (ALT/AST ratio) + BMI (+ 2, if female; +2, if presence of diabetes) [29]. An HSI value > 36 was indicative of hepatic steatosis, according to the cohort study published by Lee et al., who first developed and validated HSI against liver ultrasonography in over 10,000 Korean individuals [29]. In this cohort study, HSI had an area under the receiver-operating curve of 0.81 (95% confidence interval [CI] 0.80–0.82). At HSI < 30 or > 36 values, HSI ruled out NAFLD with a sensitivity of 92.5% (95% CI 91.4–93.5%) or detected NAFLD with a specificity of 92.4% (95% CI 91.3–93.4%). Recently, HSI was also validated against liver ultrasonography in Italian patients with T2DM [30] and against magnetic resonance imaging in adults with T1DM, showing a sensitivity of 86%, specificity of 66%, positive predictive value of 0.50, and negative predictive value of 0.92 [31]. Notably, in a subset of our participants (n = 326, 26% of total), in whom we also had data on liver ultrasonography, we performed a receiver-operating characteristic curve analysis to identify hepatic steatosis according to the HSI. The area under the receiver-operating curve (AUROC) for HSI was 0.72 (95% CI 0.63–0.75).

We have also calculated the fibrosis (FIB)-4 index by using the following formula: FIB-4 index = age × AST (IU/L)/platelet count (×10^9^/L) × √ALT (IU/L). The FIB-4 index is one of the most widely used non-invasive scores of advanced fibrosis [32]. A FIB-4 cut-off ≥ 1.3 was suggestive of significant liver fibrosis [33].

### The 10-year risk of CVD risk estimates

We used the Steno type 1 risk engine [24] and the ASCVD risk calculator [25] to estimate the 10-year risk of developing a first fatal or nonfatal CVD event. It is known that the ASCVD risk calculator is not a specific tool for people with T1DM, while the Steno type 1 risk engine is a specific risk-estimation tool for people with T1DM as it has been implemented and validated in a cohort of 4,306 Danish adults with T1DM attending the Steno Diabetes center [24]. The Steno type 1 risk engine estimates the 10-year risk of developing a first fatal or nonfatal CVD event (ischemic heart disease, ischemic stroke, heart failure, and peripheral artery disease). It includes the following 10 variables: age, sex, diabetes duration, systolic blood pressure, LDL-cholesterol, HbA1c, albuminuria, e-GFR, smoking, and regular exercise. The Steno type 1 risk engine allows the 10-year estimated CVD risk to be categorized as follows: low risk (< 10%), moderate risk (10-19.9%), and high risk (≥ 20%) [24]. The ASCVD risk calculator was proposed in 2013 by the American College of Cardiology/American Heart Association Task Force on Practice and is based on data derived from representative community-based cohorts with White and Black individuals. In particular, this risk calculator estimates the 10-year risk of developing a first hard CVD event (coronary heart disease mortality, nonfatal myocardial infarction, and fatal or nonfatal stroke) and includes the following 9 variables: age, sex, race, total cholesterol, HDL-cholesterol, systolic blood pressure, hypertension treatment, smoking, and diabetes status [25]. The ASCVD risk calculator allows the 10-year estimated CVD risk to be categorized as follows: low risk (< 5%), borderline risk (5-7.4%), intermediate risk (7.5-19.9%), and high risk (≥ 20%) [25]. For this study, we merged the borderline and intermediate CVD risk groups into a single group called the intermediate ASCVD risk group.

### Statistical analysis

Continuous variables were expressed as means ± SD or medians (interquartile ranges), and categorical variables as proportions. Differences in the main clinical and biochemical characteristics of participants stratified either by the presence of hepatic steatosis (with or without coexisting significant fibrosis) or by 10-year estimated CVD risk categories were tested by the one-way ANOVA for normally distributed continuous variables, the Kruskal-Wallis test for non-normally distributed variables, and the chi-squared test for categorical variables. Univariable and multivariable logistic regression analyses were performed to examine the associations between hepatic steatosis (with or without coexisting significant fibrosis) and the 10-year CVD risk estimates. In these logistic regression models, the dependent dichotomous variable was as follows: (a) the high or moderate Steno type 1 risk groups combined vs. the low Steno type 1 risk group; or (b) the high or intermediate ASCVD risk groups combined vs. the low ASCVD risk group. Specifically, we performed unadjusted logistic regression models and two forced-entry adjusted regression models. The first model was unadjusted; the second model was adjusted for sex, BMI, diabetes duration, HbA1c, presence of CKD (defined as e-GFR < 60 mL/min/1.73 m^2^ or abnormal albuminuria), and lipid-lowering medication use; and, finally, the third model included the same model 2’s covariates after excluding individuals with significant alcohol intake. Notably, as age was included both in the FIB-4 index and in the two CVD risk equations, and hypertension and smoking were also included in the two CVD risk equations, we decided not to include age, smoking, and hypertension status among the covariates of these multivariable regression models to reduce possible multicollinearity problems.

All statistical tests were two-sided and a *P*-value < 0.05 was considered statistically significant. Statistical analyses were performed using STATA software, version 17.0 (STATA, College Station, Texas, USA).

## Results

### Baseline characteristics

Among the 1,254 adult outpatients with established T1DM included in the study (55% men; mean [± SD] age 46 ± 14 years; diabetes duration 22 ± 12 years; BMI 25.1 ± 4.1 kg/m^2^; HbA1c 7.8 ± 1.1%), 654 (52.2%) patients had HSI ≤ 36 (i.e., indicative of absent hepatic steatosis), 509 (40.6%) had HSI > 36 and FIB-4 score < 1.3 (suggestive of steatosis without significant fibrosis) and 91 (7.3%) had HSI > 36 and FIB-4 score ≥ 1.3 (suggestive of steatosis with significant fibrosis). When we stratified participants according to low, intermediate, and high HSI values, 115 (9.2%) subjects had HSI < 30, 539 (43%) had intermediate HSI values between 30 and 36, and 600 (47.8%) had HSI > 36. Among the 91 subjects with FIB-4 index ≥ 1.3, about a third (n = 30) had FIB-4 index > 2.67 (indicative of advanced liver fibrosis). Using the Steno type 1 risk engine, 545 (43.5%) patients were classified as having a low 10-year estimated CVD risk, 359 (28.6%) had a moderate CVD risk, and 350 (27.9%) had a high CVD risk. Similarly, using the 10-year ASCVD risk calculator, 703 (56.0%) patients were classified as having a low risk, 348 (27.8%) had intermediate risk, and 203 (16.2%) had a high CVD risk.

Table 1 summarizes the main clinical and biochemical characteristics of participants stratified by the presence or absence of hepatic steatosis with or without coexisting significant fibrosis. Compared to patients with or without steatosis, those with steatosis and significant fibrosis were more likely to be older, overweight/obese, and less likely to be smokers. They also had a longer duration of diabetes, higher values of HbA1c, blood pressure, serum triglycerides, and liver enzymes, as well as lower platelet count, lower LDL-cholesterol, and lower e-GFR_CKD−EPI_. Patients with hepatic steatosis and significant fibrosis achieved more frequently a plasma LDL-cholesterol level < 1.8 mmol/L (< 70 mg/dL) (although the proportion, i.e., 15.4%, was extremely low, considering the high 10-year estimated CVD risk of this patient subgroup), and had a greater prevalence of hypertension, diabetic retinopathy of any degree, abnormal albuminuria, or CKD. The total daily insulin doses and the proportion of those treated with antihypertensive medications (i.e., diuretics, beta-blockers, calcium-channel blockers, or renin-angiotensin system inhibitors), antiplatelet or lipid-lowering agents were also higher in patients with hepatic steatosis and significant fibrosis. Sex distribution, regular exercise, daily alcohol intake, total cholesterol and glucose levels did not significantly differ among the patient groups. None of these patients with T1DM were treated with metformin or other glucose-lowering agents in addition to insulin treatment.

**Table 1:** Clinical and biochemical characteristics of adults with type 1 diabetes, stratified by the presence of hepatic steatosis with or without coexisting significant liver fibrosis (non-invasively assessed by HSI and FIB-4 scores)

	Patients with HSI ≤ 36 (n = 654)	Patients with HSI > 36 and FIB4 < 1.3 (n = 509)	Patients with HSI > 36 and FIB4 ≥ 1.3 (n = 91)	*P*-value
Age (years)	45 ± 16	44 ± 13	63 ± 12	< 0.001
Male sex (%)	55.1	56.6	47.3	0.258
BMI (kg/m^2^)	22.7 ± 2.3	27.9 ± 4.0	29.3 ± 4.7	< 0.001
Current smokers (%)	29.4	22.6	13.2	< 0.001
Regular physical exercise (≥ 3.5 h/week) (%)	48.3	46.6	35.2	0.061
Alcohol intake (%)	18.2	14.7	12.1	0.251
Diabetes duration (years)	22 ± 12	21 ± 11	30 ± 14	< 0.001
Glucose (mg/dL)	175 ± 72	185 ± 70	178 ± 68	0.242
HbA1c (%)	7.7 ± 1.2	7.9 ± 1.3	8.2 ± 1.0	< 0.001
Systolic blood pressure (mmHg)	126 ± 18	129 ± 16	140 ± 20	< 0.001
Diastolic blood pressure (mmHg)	75 ± 8	77 ± 9	78 ± 10	< 0.001
Total cholesterol (mg/dL)	180 ± 34	184 ± 33	179 ± 43	0.167
HDL cholesterol (mg/dL)	61 ± 16	55 ± 14	50 ± 18	< 0.001
LDL cholesterol (mg/dL)	103 ± 28	108 ± 29	99 ± 36	0.001
LDL cholesterol < 70 mg/dL (%)	10.1	6.7	15.4	0.012
Triglycerides (mg/dL)	70 (56–95)	83 (62–120)	89 (66–131)	< 0.001
AST (IU/L)	16 (12–22)	17 (12–28)	24 (15–30)	< 0.001
ALT (IU/L)	17 (13–22)	21 (16–29)	22 (17–31)	< 0.001
GGT (IU/L)	15 (11–21)	18 (13–29)	21 (14–40)	< 0.001
Platelet count (x 100,000/mm^3^)	241 ± 68	258 ± 63	191 ± 57	< 0.001
Creatinine (mg/dL)	0.9 ± 0.3	0.9 ± 0.5	1.1 ± 0.9	< 0.001
eGFR_CKD−EPI_ (mL/min/1.73 m^2^)	98 ± 20	97 ± 19	79 ± 26	< 0.001
Abnormal albuminuria (%)	11.3	13.9	30.8	< 0.001
CKD (%)	13.5	14.9	37.4	< 0.001
Diabetic retinopathy (%)	27.2	33.7	51.1	< 0.001
Hypertension (%)	32.9	39.9	74.7	< 0.001
Total daily insulin dose (IU/day)	38 ± 15	49 ± 20	45 ± 17	< 0.001
Antiplatelet drug users (%)	11.5	10.8	39.6	< 0.001
Diuretic users (%)	5.5	9.3	37.4	< 0.001
Beta-blocker users (%)	5.5	6.1	26.4	< 0.001
Calcium-channel blocker users (%)	4.4	7.5	24.2	< 0.001
ACE-i/ARB users (%)	24.2	33.2	67.0	< 0.001
Statin users (%)	27.4	33.9	62.6	< 0.001

Cohort size: *n* = 1,254. Data are expressed as means ± SD, medians, and interquartile ranges (IQRs) or percentages. Differences among the three patient groups were tested by the Chi-squared test for categorical variables, the one-way ANOVA for normally distributed continuous variables, and the Kruskal-Wallis test for non-normally distributed variables (i.e., serum liver enzymes and triglycerides)

Note: Alcohol intake was defined as ≥ 2 alcohol units per day in men and ≥ 1 alcohol unit per day in women, respectively. Abnormal albuminuria was defined as urine albumin-to-creatinine ratio ≥ 3.0 mg/mmol. CKD was defined as eGFR_CKD−EPI_ <60 mL/min/1.73 m^2^ or abnormal albuminuria.

Abbreviations: ACE, angiotensin-converting-enzyme inhibitor; ARB, angiotensin II receptor blocker; ALT, alanine aminotransferase; AST, aspartate aminotransferase; BMI, body mass index; CKD, chronic kidney disease; eGFR_CKD−EPI_, estimated glomerular filtration rate calculated by the CKD-Epidemiology Collaboration study equation; GGT, gamma-glutamyl-transferase; FIB-4, fibrosis 4; HSI, hepatic steatosis index

Table 2 shows the main clinical and biochemical characteristics of participants stratified by 10-year estimated CVD risk categories using the Steno type 1 risk engine. Patients with a high 10-year estimated CVD risk were more likely to be older, overweight/obese, and less likely to be engaged in regular physical activity than those with low or moderate CVD risk. The former also had longer diabetes duration, higher values of HbA1c, blood pressure, serum triglycerides, total cholesterol, liver enzymes, and lower platelet count and e-GFR_CKD−EPI_. Compared to the other two patient subgroups, patients at high 10-year estimated CVD risk also had a greater prevalence of hypertension, diabetic retinopathy of any degree, abnormal albuminuria, and CKD. Furthermore, the proportion of patients treated with antihypertensive, antiplatelet, or lipid-lowering agents was greater among those at high CVD risk. Sex distribution, smoking history, and daily alcohol intake, as well as total daily insulin doses and the proportion of those achieving a plasma LDL-cholesterol level < 1.8 mmol/L did not significantly differ among the patient groups.

**Table 2:** Clinical and biochemical characteristics of patients with type 1 diabetes, stratified by categories of the Steno type 1 risk engine

	Patients with low CVD risk (n = 545)	Patients with intermediate CVD risk (n = 359)	Patients with high CVD risk (n = 350)	*P*-value
Age (years)	33 ± 8	49 ± 7	64 ± 10	< 0.001
Male sex (%)	55.2	57.7	52.3	0.355
BMI (kg/m^2^)	24.6 ± 3.9	25.5 ± 4.1	26.2 ± 5.0	< 0.001
Current smokers (%)	27.9	25.6	21.4	0.095
Regular physical exercise (≥ 3.5 h/week) (%)	56.9	46.2	31.1	< 0.001
Alcohol intake (%)	15.7	17.5	15.7	0.138
Diabetes duration (years)	16 ± 8	22 ± 11	31 ± 13	< 0.001
Glucose (mg/dL)	175 ± 71	175 ± 69	187 ± 72	0.149
HbA1c (%)	7.7 ± 1.1	7.9 ± 1.2	8.2 ± 1.4	< 0.001
Systolic blood pressure (mmHg)	120 ± 14	127 ± 14	141 ± 19	< 0.001
Diastolic blood pressure (mmHg)	75 ± 9	77 ± 9	77 ± 10	< 0.001
Total cholesterol (mg/dL)	177 ± 33	186 ± 33	185 ± 36	< 0.001
HDL cholesterol (mg/dL)	56 ± 14	60 ± 15	61 ± 18	< 0.001
LDL cholesterol (mg/dL)	104 ± 27	108 ± 29	103 ± 32	0.064
LDL cholesterol < 70 mg/dL (%)	9.0	7.8	10.6	0.436
Triglycerides (mg/dL)	71 (55–103)	74 (59–102)	85 (65–119)	< 0.001
AST (IU/L)	20 ± 9	22 ± 9	25 ± 15	< 0.001
ALT (IU/L)	17 (12–23)	19 (12–26)	23 (12–28)	0.001
GGT (IU/L)	15 (11–20)	17 (12–26)	20 (14–36)	< 0.001
Platelet count (x 100,000/mm^3^)	244 ± 63	248 ± 66	241 ± 76	0.337
Creatinine (mg/dL)	0.8 ± 0.2	0.8 ± 0.2	1.0 ± 0.8	< 0.001
eGFR_CKD−EPI_ (mL/min/1.73 m^2^)	108 ± 14	96 ± 13	79 ± 22	< 0.001
Abnormal albuminuria (%)	4.2	9.8	32.9	< 0.001
CKD (%)	4.2	10.0	39.7	< 0.001
Diabetic retinopathy (%)	17.7	34.9	49.6	< 0.001
Hypertension (%)	14.9	39.6	75.1	< 0.001
Total daily insulin dose (IU/day)	43 ± 18	43 ± 17	42 ± 20	0.629
Antiplatelet drug users (%)	2.0	8.7	35.5	< 0.001
Diuretic users (%)	1.7	6.7	24.0	< 0.001
Beta-blocker users (%)	1.7	6.2	17.1	< 0.001
Calcium-channel blocker users (%)	0.9	5.0	18.9	< 0.001
ACE-i/ARB users (%)	7.2	32.3	66.6	< 0.001
Statin users (%)	8.3	37.3	65.7	< 0.001

Cohort size: *n* = 1,254. Data are expressed as means ± SD, medians, and interquartile ranges (IQRs) or percentages. Differences among the three patient groups were tested by the Chi-squared test for categorical variables, the one-way ANOVA for normally distributed continuous variables, and the Kruskal-Wallis test for non-normally distributed variables (i.e., serum liver enzymes and triglycerides)

Note: Alcohol intake was defined as ≥ 2 alcohol units per day in men and ≥ 1 alcohol unit per day in women, respectively. Abnormal albuminuria was defined as urine albumin-to-creatinine ratio ≥ 3.0 mg/mmol. CKD was defined as eGFR_CKD−EPI_ <60 mL/min/1.73 m^2^ or abnormal albuminuria.

Abbreviations: ACE, angiotensin-converting-enzyme inhibitor; ARB, angiotensin II receptor blocker; ALT, alanine aminotransferase; AST, aspartate aminotransferase; BMI, body mass index; CKD, chronic kidney disease; eGFR_CKD−EPI_, estimated glomerular filtration rate calculated by the CKD-Epidemiology Collaboration study equation; GGT, gamma-glutamyl-transferase; FIB-4, fibrosis 4; HSI, hepatic steatosis index.

Table 3 shows the main clinical and biochemical characteristics of participants stratified by 10-year estimated CVD risk categories using the ASCVD risk calculator. Compared to those at low or intermediate risk, patients at high 10-year estimated CVD risk were more likely to be older, men, overweight/obese, and less likely to be engaged in regular physical activity. In addition, patients at high CVD risk also had longer diabetes duration, higher blood pressure and serum liver enzymes, lower platelet count, and lower e-GFR_CKD−EPI_. These patients also had a higher prevalence of hypertension, retinopathy of any degree, abnormal albuminuria, and CKD. The proportion of those treated with antihypertensive, antiplatelet drugs, or lipid-lowering agents was also higher in patients at high 10-year estimated CVD risk. Alcohol intake, HbA1c, glucose, HDL-cholesterol, and total daily insulin doses did not significantly differ among the patient groups.

**Table 3:** Clinical and biochemical characteristics of patients with type 1 diabetes, stratified by 10-year ASCVD risk calculator categories

	Patients with low CVD risk (n = 703)	Patients with intermediate CVD risk (n = 348)	Patients with high CVD risk (n = 203)	*P*-value
Age (years)	37 ± 10	52 ± 10	66 ± 13	< 0.001
Male sex (%)	50.0	61.9	57.7	< 0.001
BMI (kg/m^2^)	24.7 ± 3.9	25.9 ± 4.8	25.8 ± 4.4	< 0.001
Current smokers (%)	20.3	38.1	25.0	< 0.001
Regular physical exercise (≥ 3.5 h/week) (%)	50.4	46.5	32.3	< 0.001
Alcohol intake (%)	13.9	18.4	15.5	0.318
Diabetes duration (years)	19 ± 10	23 ± 13	29 ± 15	< 0.001
Glucose (mg/dL)	175 ± 73	181 ± 70	183 ± 67	0.499
HbA1c (%)	7.8 ± 1.2	8.0 ± 1.5	7.9 ± 1.0	0.052
Systolic blood pressure (mmHg)	121 ± 14	132 ± 17	143 ± 19	< 0.001
Diastolic blood pressure (mmHg)	75 ± 9	78 ± 9	77 ± 10	< 0.001
Total cholesterol (mg/dL)	178 ± 31	190 ± 36	182 ± 37	< 0.001
HDL cholesterol (mg/dL)	59 ± 15	57 ± 17	59 ± 18	0.206
LDL cholesterol (mg/dL)	103 ± 25	109 ± 31	104 ± 36	0.001
LDL cholesterol < 70 mg/dL (%)	9.4	7.2	11.3	0.242
Triglycerides (mg/dL)	68 (55–94)	87 (65–124)	84 (64–112)	< 0.001
AST (IU/L)	20 ± 10	23 ± 10	25 ± 16	< 0.001
ALT (IU/L)	21 ± 15	24 ± 14	23 ± 16	0.004
GGT (IU/L)	15 (11–20)	20 (14–32)	21 (14–38)	< 0.001
Platelet count (x 100,000/mm^3^)	248 ± 68	246 ± 71	233 ± 71	0.019
Creatinine (mg/dL)	0.80 (0.70–0.90)	0.84 (0.72–0.95)	0.81 (0.70-1.00)	0.001
eGFR_CKD−EPI_ (mL/min/1.73 m^2^)	104 ± 17	91 ± 20	81 ± 22	< 0.001
Abnormal albuminuria (%)	8.0	17.9	28.5	< 0.001
CKD (%)	7.9	20.0	34.1	< 0.001
Diabetic retinopathy (%)	24.6	38.1	43.9	< 0.001
Hypertension (%)	20.6	51.5	78.2	< 0.001
Total daily insulin dose (IU/day)	42 ± 17	44 ± 19	41 ± 18	0.117
Antiplatelet drug users (%)	2.7	17.1	39.3	< 0.001
Diuretic users (%)	2.6	12.3	26.8	< 0.001
Beta-blocker users (%)	3.2	8.8	17.7	< 0.001
Calcium-channel blocker users (%)	2.0	8.0	25.0	< 0.001
ACE-i/ARB users (%)	13.5	44.0	64.1	< 0.001
Statin users (%)	14.7	49.1	66.5	< 0.001

Cohort size: *n* = 1,254. Data are expressed as means ± SD, medians and interquartile ranges (IQRs) or percentages. Differences among the three patient groups were tested by the Chi-squared test for categorical variables, the one-way ANOVA for normally distributed continuous variables, and the Kruskal-Wallis test for non-normally distributed variables (i.e., serum liver enzymes, creatinine, and triglycerides)

Note: Alcohol intake was defined as ≥ 2 alcohol units per day in men and ≥ 1 alcohol unit per day in women, respectively. Abnormal albuminuria was defined as urine albumin-to-creatinine ratio ≥ 3.0 mg/mmol. CKD was defined as eGFR_CKD−EPI_ <60 mL/min/1.73 m^2^ or abnormal albuminuria.

Abbreviations: ACE, angiotensin-converting-enzyme inhibitor; ARB, angiotensin II receptor blocker; ALT, alanine aminotransferase; AST, aspartate aminotransferase; BMI, body mass index; CKD, chronic kidney disease; eGFR_CKD−EPI_, estimated glomerular filtration rate calculated by the CKD-Epidemiology Collaboration study equation; GGT, gamma-glutamyl-transferase; FIB-4, fibrosis 4; HSI, hepatic steatosis index.

### The 10-year CVD risk estimates in NAFLD with or without significant fibrosis

**Fig. 1:**
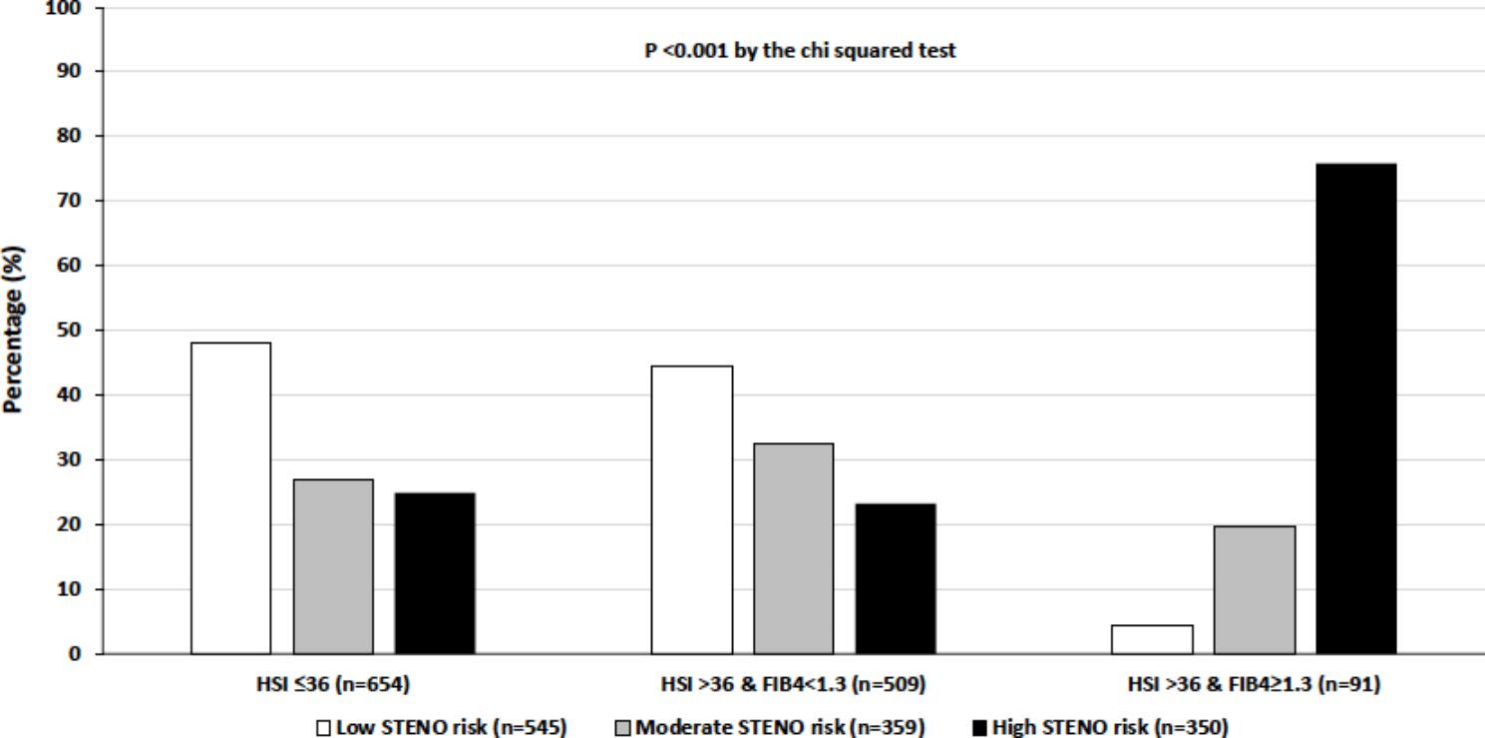
Prevalence rates of the 10-year risk of developing a first fatal or nonfatal CVD (as estimated by the Steno type 1 risk engine) in adults with T1DM stratified by the presence or absence of hepatic steatosis with or without coexisting significant fibrosis (as determined by HSI and FIB-4 scores)

**Fig. 2:**
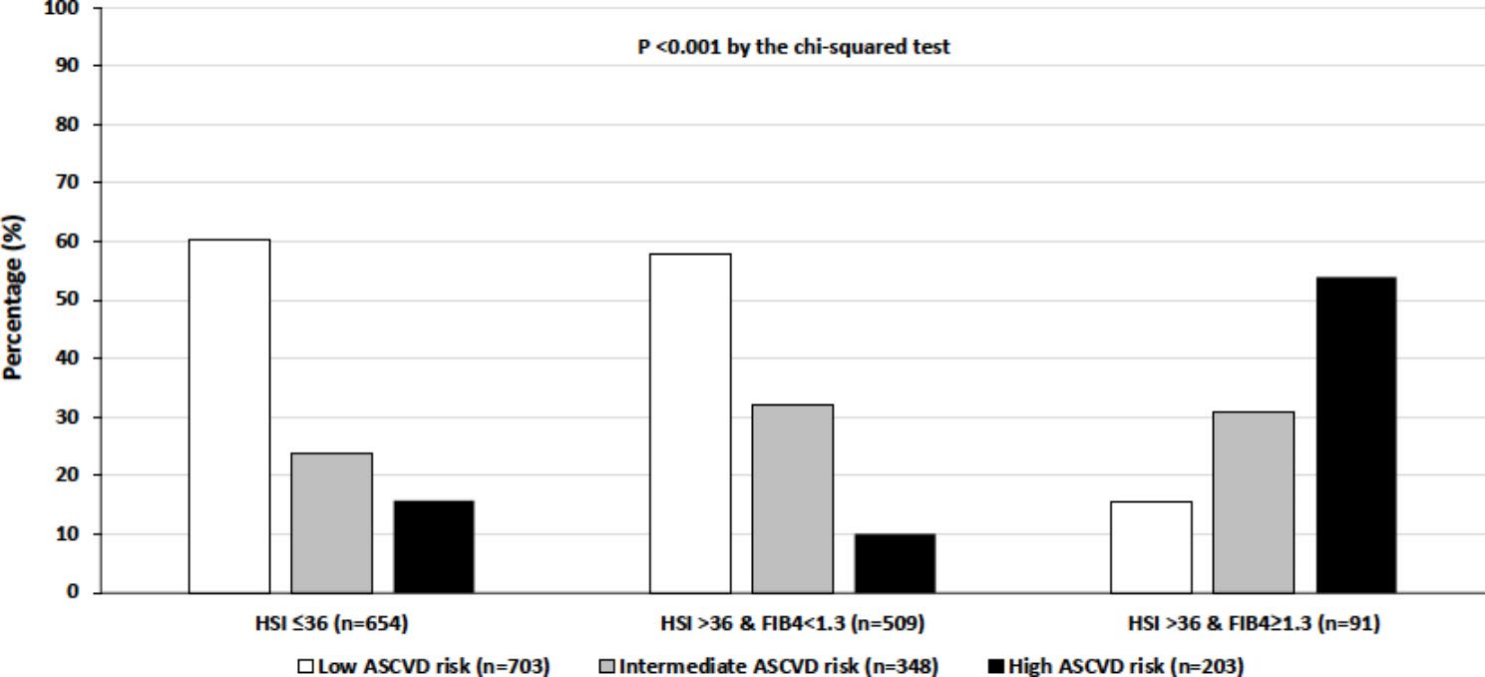
Prevalence rates of the 10-year risk of developing a first fatal or nonfatal CVD (as estimated by the ASCVD risk calculator) in adults with T1DM stratified by the presence or absence of hepatic steatosis with or without coexisting significant fibrosis (as determined by HSI and FIB-4 scores)

We calculated the prevalence rates of the 10-year estimated CVD risk categories, using the Steno type 1 risk engine (Fig. 1) or the ASCVD risk calculator (Fig. 2), in participants stratified by the presence or absence of hepatic steatosis with or without coexisting significant fibrosis. Using the Steno type 1 risk engine, we found that a remarkably higher proportion of patients with steatosis and significant fibrosis had a high 10-year estimated risk of developing a first fatal or nonfatal CVD event compared to their counterparts with steatosis alone or without steatosis (75.8% vs. 23.2% vs. 24.9%, p < 0.001 by the chi-squared test). Similarly, using the ASCVD risk calculator, we found that a significantly greater proportion of patients with steatosis and significant fibrosis had a high 10-year estimated CVD risk compared to those with steatosis alone or without steatosis (53.9% vs. 10.0% vs. 15.8%, p < 0.001).

We performed subgroup analyses by median BMI values (< 24.8 vs. ≥24.8 kg/m^2^) (Supplementary Fig. 2), by median diabetes duration (< 20 vs. ≥20 years) (Supplementary Fig. 3), by median age (< 45 vs. ≥45 years) (Supplementary Fig. 4), by sex (Supplementary Fig. 5), or by hypertension status (Supplementary Fig. 6). These subgroup analyses confirmed that the 10-year estimated CVD risk (as calculated by the Steno type 1 risk engine or the ASCVD risk calculator) was remarkably greater in patients with hepatic steatosis and significant fibrosis than in those with steatosis alone or without steatosis, in both sexes and all other patient subgroups considered. Similar results were also found when we stratified our participants by median HbA1c (< 7.7% vs. ≥7.7%) or smoking status (data not shown).

Table 4 shows the association between hepatic steatosis (with or without coexisting significant fibrosis) and the 10-year estimated CVD risk. In unadjusted regression models, patients with hepatic steatosis and significant fibrosis had a ~ 8-fold (for the ASCVD risk calculator) to ~ 20-fold (for the Steno type 1 risk engine) increased risk of having a high/moderate 10-year estimated CVD risk when compared to their counterparts without steatosis. Notably, this risk remained significant even after adjustment for sex, BMI, diabetes duration, HbA1c, presence of CKD, and lipid-lowering medication use (adjusted model 1). Conversely, the 10-year estimated CVD risk was comparable between patients with steatosis alone and those without steatosis. Excluding participants (n = 211) with estimated alcohol intake ≥ 2 alcohol units per day for men and ≥ 1 alcohol unit per day for women did not affect the results (adjusted model 2). In these multivariable logistic regression models, other variables that were independently associated with an increased 10-year estimated CVD risk were longer diabetes duration, CKD, and non-use of statins (for both CVD prediction models), as well as male sex (for the ASCVD risk calculator) and higher HbA1c (for the Steno type 1 risk engine) (p < 0.001 for all).

**Table 4:** Association between hepatic steatosis with or without coexisting significant fibrosis and the 10-year estimated CVD risk (using the Steno type 1 risk engine or the ASCVD risk calculator)

Logistic Regression Analyses	Odds Ratios (95% confidence intervals)	*P*-value
**Y = High or moderate risk vs. low Steno type 1 risk score**		
*Unadjusted model*		
Patients with HSI ≤ 36 (n = 654)	Ref.	-
Patients with HSI > 36 and FIB4 < 1.3 (n = 509)	1.16 (0.92–1.47)	0.202
Patients with HSI > 36 and FIB4 ≥ 1.3 (n = 91)	20.2 (7.33–55.7)	< 0.001
*Adjusted model 1*		
Patients with HSI ≤ 36 (n = 654)	Ref.	-
Patients with HSI > 36 and FIB4 < 1.3 (n = 509)	0.94 (0.65–1.36)	0.753
Patients with HSI > 36 and FIB4 ≥ 1.3 (n = 91)	11.4 (3.54–36.9)	< 0.001
*Adjusted model 2* (n = 1,043)		
Patients with HSI ≤ 36 (n = 532)	Ref.	-
Patients with HSI > 36 and FIB4 < 1.3 (n = 432)	0.79 (0.52–1.18)	0.244
Patients with HSI > 36 and FIB4 ≥ 1.3 (n = 79)	10.9 (2.99–39.9)	< 0.001
**Y = High or intermediate risk**vs.**low ASCVD risk score**		
*Unadjusted model*		
Patients with HSI ≤ 36 (n = 654)	Ref.	-
Patients with HSI > 36 and FIB4 < 1.3 (n = 509)	1.12 (0.88–1.41)	0.364
Patients with HSI > 36 and FIB4 ≥ 1.3 (n = 91)	8.38 (4.64–15.1)	< 0.001
*Adjusted model 1*		
Patients with HSI ≤ 36 (n = 654)	Ref.	-
Patients with HSI > 36 and FIB4 < 1.3 (n = 509)	0.99 (0.71–1.39)	0.982
Patients with HSI > 36 and FIB4 ≥ 1.3 (n = 91)	4.83 (2.39–9.78)	< 0.001
*Adjusted model 2* (n = 1,043)		
Patients with HSI ≤ 36 (n = 532)	Ref.	-
Patients with HSI > 36 and FIB4 < 1.3 (n = 432)	0.93 (0.64–1.36)	0.697
Patients with HSI > 36 and FIB4 ≥ 1.3 (n = 79)	6.93 (3.00-15.9)	< 0.001

Cohort size, n = 1,254, except where indicated. Data are expressed as odds ratio (OR) and 95% confidence interval, assessed by univariable and multivariable logistic regression analyses. The dependent variable of logistic regression models was: (a) the high or moderate Steno type 1 risk groups combined vs. the low Steno type 1 risk group, or (b) the high or Intermediate ASCVD risk groups combined vs. the low ASCVD risk group. Regression model 1 was adjusted for sex, BMI, diabetes duration, HbA1c, presence of CKD (defined as e-GFR < 60 mL/min/1.73 m^2^ or abnormal albuminuria), and lipid-lowering medication use. Regression model 2 was adjusted for the same model’s 1 covariates after excluding those with significant alcohol intake (n = 211)

As sensitivity analyses, we also repeated the above-mentioned logistic regression models after excluding participants with intermediate HSI values (i.e., those with HSI ranging from 30 to 36) from the analysis (Supplementary Table 1) or after including these participants among those with hepatic steatosis (in such case, the diagnosis of NAFLD was defined as HSI > 30, instead of HSI > 36) (Supplementary Table 2). Notably, the results of the study remained unchanged.

## Discussion

The main and novel findings of our large multicenter cross-sectional study that included 1,254 Italian adult outpatients with T1DM without pre-existing CVD (i.e., subjects in primary prevention of CVD) are as follows: (a) the 10-year estimated risk of developing a first fatal or nonfatal CVD event was markedly greater in patients with hepatic steatosis and significant fibrosis compared to those with steatosis alone or without steatosis; (b) this CVD risk remained statistically significant even after adjusting for sex, BMI, diabetes duration, HbA1c, CKD, and lipid-lowering medication use; and (c) subgroups analyses confirmed that the 10-year estimated CVD risk was greater in those with hepatic steatosis and liver fibrosis compared to the other two patient subgroups, regardless of age, sex, BMI, diabetes duration, HbA1c, smoking history, and hypertension status.

This is the most updated and largest cross-sectional study aimed at examining the association between NAFLD (with and without coexisting significant fibrosis) and the 10-year CVD risk estimates in adults with T1DM.

It is well known that NAFLD is a growing public health problem in people with T2DM, causing considerable healthcare costs, economic losses, and reduced health-related quality of life [1]. An updated meta-analysis reported a strong association between NAFLD and an increased risk of developing CVD events, i.e., the predominant cause of death in people with NAFLD [9]. To date, the clinical burden of NAFLD in people with T1DM has little been studied. There are few data (mostly derived from small single-center studies) regarding the magnitude of the clinical burden of NAFLD in adults with T1DM [20–23], especially regarding its possible adverse effects on CVD risk. For instance, in 2010, in a small cross-sectional study of 250 Italian outpatients with T1DM, Targher et al. [21]. reported for the first time that ultrasound-detected NAFLD (present in ~ 45% of patients) was associated, independently of common CVD risk factors, with the presence of asymptomatic/symptomatic CVD (assessed by patient history, chart review, electrocardiogram, and echo-Doppler scanning of carotid and lower limb arteries). In 2012, in a subsequent cross-sectional study of 343 Italian adult outpatients with T1DM (~ 52% of whom had NAFLD on ultrasonography), Targher et al. [22] reported that NAFLD was associated with an increased prevalence of asymptomatic/symptomatic coronary, cerebrovascular and peripheral vascular disease (adjusted-odds ratio 7.6, 95% CI 3.6–24.0), independent of multiple CVD risk factors. In 2016, in a single-center cross-sectional study of 722 Chinese adults with T1DM, Zhang et al. [20] reported that NAFLD on ultrasonography was associated with increased carotid-artery intimal medial thickness and an increased prevalence of carotid atherosclerotic plaques, independent of traditional CVD factors. Finally, and most interestingly, in a small retrospective longitudinal study involving 286 adult patients with T1DM followed for a mean period of 5.3 years, Mantovani et al. [23] reported that NAFLD on ultrasonography (present in ~ 52% of patients) was associated with an increased incidence of CVD events, independently of traditional CVD risk factors and diabetes-related variables.

Collectively, therefore, the findings of our large multicenter cross-sectional study corroborate and expand the results of the previously published studies, showing that T1DM patients with NAFLD and significant fibrosis had a remarkably higher 10-year estimated risk of developing a first fatal or nonfatal CVD event compared to those with hepatic steatosis alone or without steatosis. Notably, the sample size of our study was at least ~ 3–4 times greater than that of the previously published studies. Furthermore, this is the first large study to examine the association between the FIB-4 index (i.e., a reliable non-invasive biomarker of advanced liver fibrosis) and the CVD risk in people with T1DM. In the previously published studies, there was no information about the severity of liver fibrosis, which is one of the strongest predictors of all-cause mortality and adverse clinical outcomes in NAFLD [6, 8, 34].

### Potential clinical implications

The findings of our study may have important clinical implications, as they support the assertion that the non-invasive identification of hepatic steatosis with coexisting significant fibrosis in individuals with T1DM can help to identify a subset of subjects at higher 10-year risk of developing a first fatal or nonfatal CVD event. Therefore, in the era of precision medicine, if further confirmed in large prospective studies on “hard” CVD endpoints, our findings point to the presence of hepatic steatosis with significant fibrosis as a possible CVD risk enhancer allowing to identify individuals with T1DM who might benefit the most from a more intensive control of the main modifiable CVD risk factors. In this regard, a complementary and interesting finding of our study was that the proportion of patients with T1DM who achieved a plasma LDL-cholesterol level < 1.8 mmol/L (i.e., a cut-off value strongly recommended by professional organizations in people with high-risk CVD without pre-existing CVD) [35, 36] was low (achieved in only 15.4%) among patients with hepatic steatosis and significant fibrosis, who were at increased 10-year estimated CVD risk. This finding further suggests that the true CVD risk of patients with T1DM is largely underappreciated in clinical practice, and statin therapy is often under-prescribed in this patient population.

### Putative mechanisms underpinning the association between NAFLD and CVD risk

Growing clinical evidence supports that NAFLD is not an “innocent” bystander of CVD but may actively contribute to its pathogenesis [9, 37–40]. The magnitude of the CVD risk parallels the underlying severity of NAFLD (especially the level of liver fibrosis), thereby resulting in the worsening of systemic/hepatic insulin resistance, increased production of atherogenic lipids, and systemic release of multiple proinflammatory, prothrombogenic, and vasoactive mediators [9, 37–40]. All these NAFLD-related mediators may adversely influence the risk of CVD, thereby contributing to the development and progression of CVD complications in people with NAFLD [9, 37–40].

### Study limitations and strengths

The current study has some important limitations. First, the cross-sectional design of the study does not allow us to establish any cause-and-effect relationships between NAFLD with significant fibrosis and the high 10-year estimated CVD risk. Second, we diagnosed hepatic steatosis using the HSI index (i.e., HSI > 36 vs. HSI ≤ 36) and not liver ultrasonography. Similarly, we used the FIB-4 index for non-invasively detecting significant fibrosis (FIB-4 index ≥ 1.3) and not vibration-controlled transient elastography (FibroScan®). In clinical practice, liver ultrasonography and FibroScan® are the two first-line imaging methods to detect hepatic steatosis and fibrosis non-invasively [41]. However, these two imaging methodologies are expensive and not easily applied in large epidemiological studies like this. That said, HSI showed a good performance in identifying hepatic steatosis when compared with liver ultrasonography or magnetic resonance imaging in adults of the general population [29], individuals with T2DM [29], and those with T1DM [31]. Furthermore, as reported in the Methods section above, we also found a satisfactory diagnostic performance of HSI (AUROC of 0.72, 95% CI 0.63–0.75) in identifying hepatic steatosis on ultrasonography in a subset of our participants. Although simple, inexpensive, and widely available serum biomarkers, such as HSI and FIB-4 scores, can be used as first-line tools, further studies using liver imaging methods for assessing hepatic steatosis and fibrosis are certainly needed to validate our findings in large cohorts of adults with T1DM. Future prospective cohort studies are also needed to confirm if NAFLD (with varying levels of fibrosis) increases the long-term risk of CVD outcomes in people with T1DM. Finally, we cannot exclude that other unmeasured factors might partly explain the observed associations.

Despite these limitations, our study has important strengths, such as the large sample size, the multicenter study design, the completeness of the database, and the exclusion of patients with important comorbidities (such as, for example, active cancer, cirrhosis and prior history of ischemic heart disease or stroke), as we believe that the inclusion of patients with such comorbidities might have confounded the interpretation of data.

## Conclusions

The results of this large multicenter cross-sectional study involving individuals with T1DM without pre-existing CVD showed that NAFLD with liver fibrosis (assessed by validated non-invasive biomarkers) was significantly associated with an increased 10-year estimated risk of developing a first fatal or nonfatal CVD event. This association remained significant even after adjusting for common CVD risk factors, diabetes-related variables, and other potential confounders. Further research is required to corroborate these findings in other cohorts of individuals with T1DM from different countries and to better elucidate whether the non-invasive detection of NAFLD with varying levels of liver fibrosis could improve CVD risk prediction in people with T1DM. In the meantime, we believe that using CVD risk prediction models is an important step to support the clinical decision on the primary prevention of CVD in people with T1DM. In addition, a multidisciplinary, team-based approach to treating individuals with T1DM and advanced NAFLD, based on a careful evaluation of related risk factors and monitoring for liver and CVD complications, is warranted.

### Electronic supplementary material

Below is the link to the electronic supplementary material.

Supplementary Material 1

## Data Availability

The dataset supporting the conclusions of this article is included within the article (and its additional files).
