# Supplementary material for: Hepatic steatosis with significant fibrosis is associated with an increased 10-year estimated risk of cardiovascular disease in adults with type 1 diabetes mellitus
Source: Cardiovasc Diabetol. 2023 Aug 10;22:204. doi: 10.1186/s12933-023-01945-x (PMC10416459; doi:10.1186/s12933-023-01945-x)
Supplement: Supplementary file 1 — Supplementary Material 1 [file 12933_2023_1945_MOESM1_ESM.pdf]

ONLINE-ONLY SUPPLEMENTARY MATERIAL

Supplementary Figure 1. The flowchart of the study.

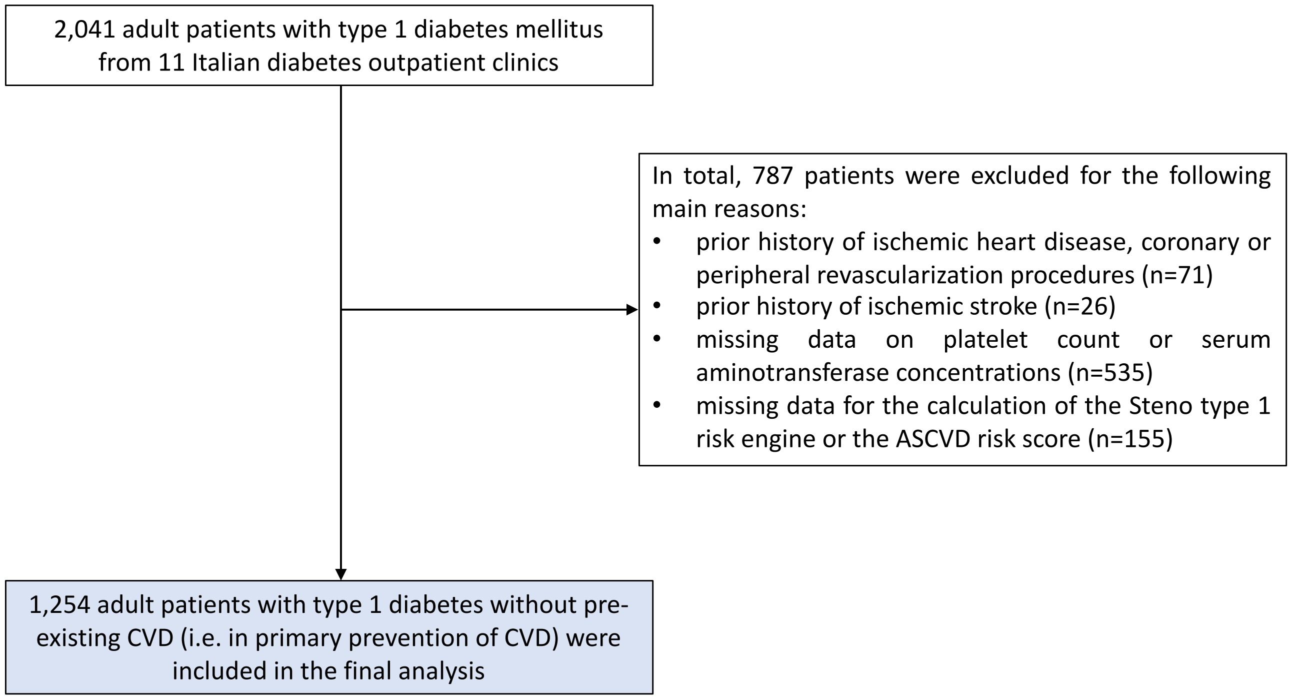

Supplementary Figure 2. Prevalence rates of the 10-year estimated CVD risk, using either the Steno type 1 risk engine (panels A and B) or the ASCVD risk calculator (panels C and D), in adult patients with T1DM who were simultaneously stratified by median BMI values (<24.8 vs. ≥24.8 kg/m^2) and presence or absence of hepatic steatosis with or without coexisting significant fibrosis. P-values are tested by the chi-squared test.

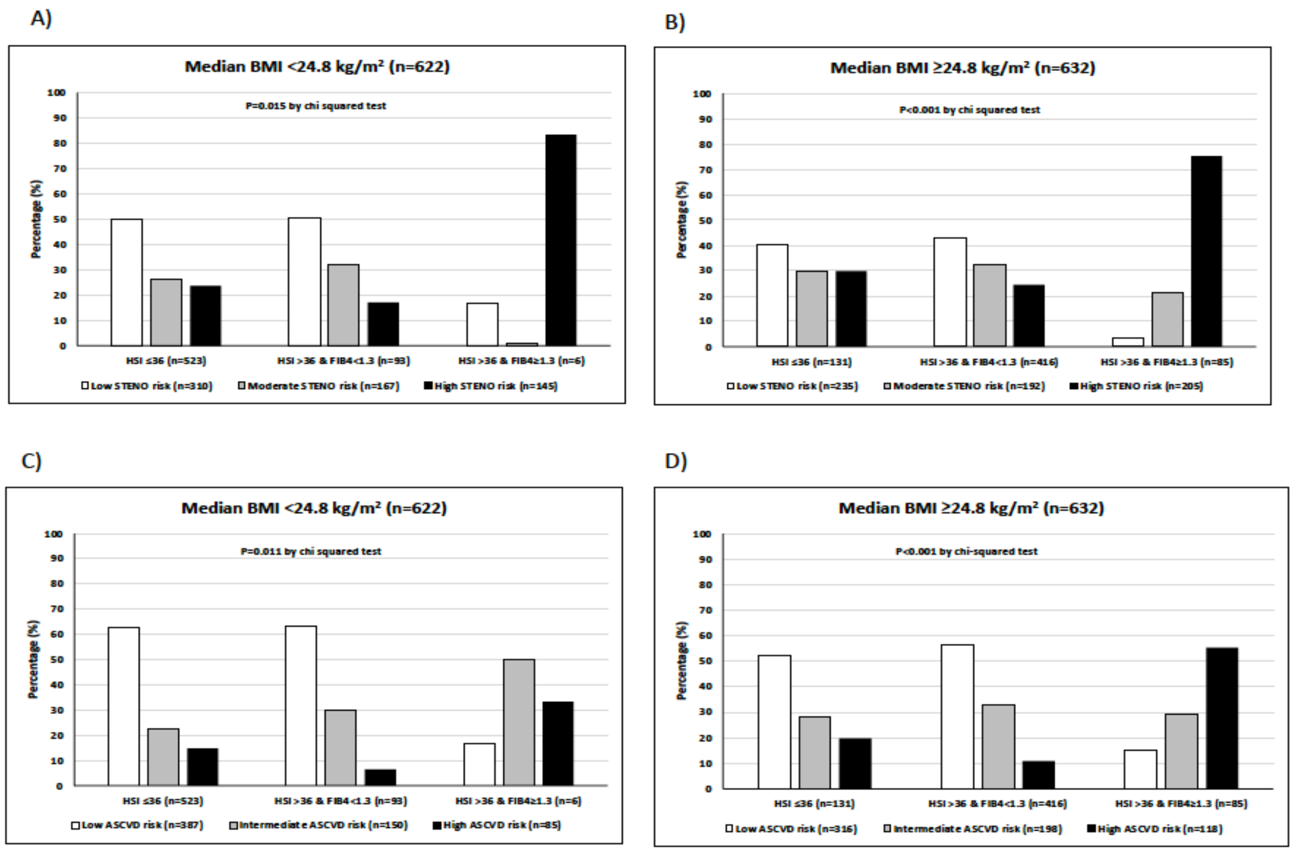

Supplementary Figure 3. Prevalence rates of the 10-year estimated CVD risk, using either the Steno type 1 risk engine (panels A and B) or the ASCVD risk calculator (panels C and D), in adult patients with T1DM who were simultaneously stratified by median duration of diabetes (<20 vs. ≥20 years) and presence or absence of hepatic steatosis with or without coexisting significant fibrosis. P-values are tested by the chi-squared test.

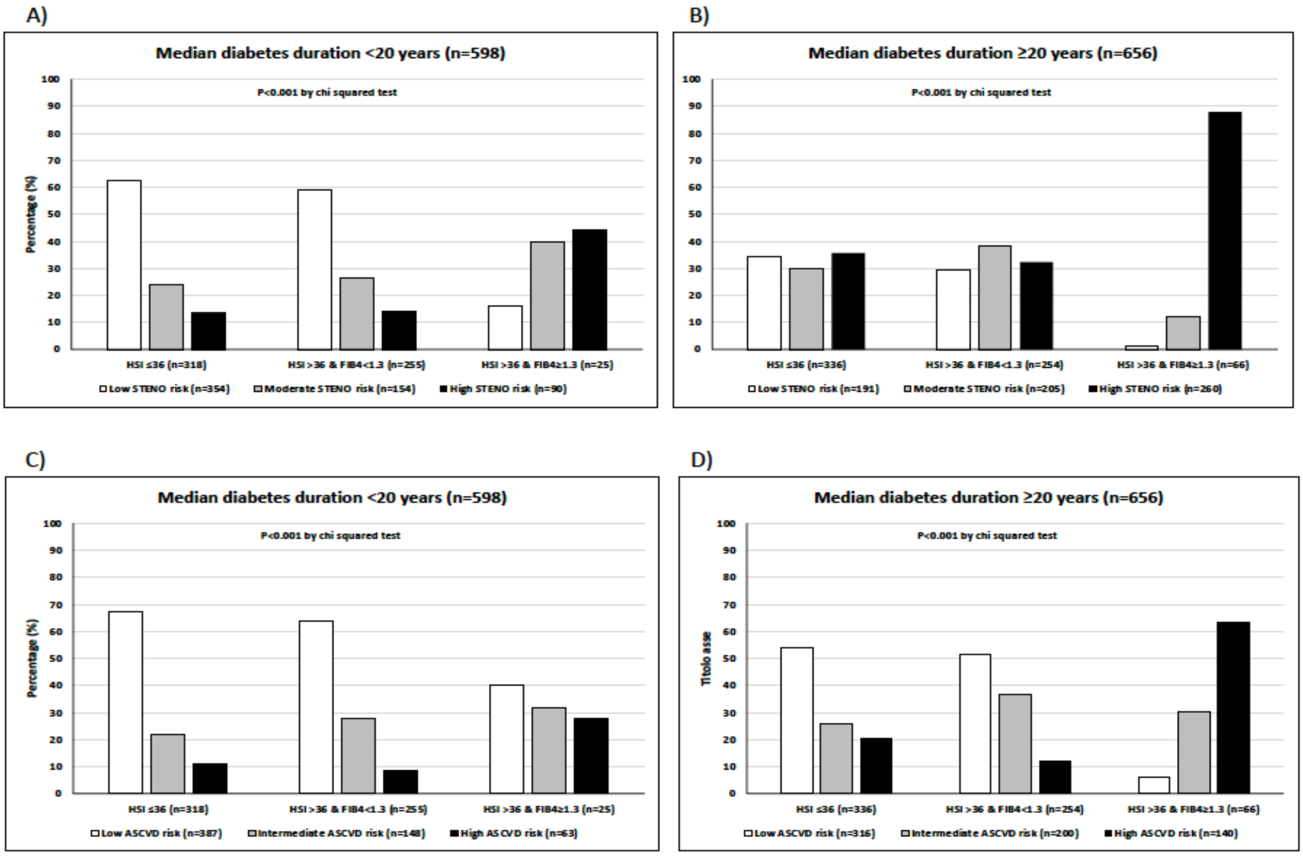

Supplementary Figure 4. Prevalence rates of the 10-year estimated CVD risk, using either the Steno type 1 risk engine (panels A and B) or the ASCVD risk calculator (panels C and D), in adult patients with T1DM who were simultaneously stratified by median age (<45 vs. ≥45 years) and presence or absence of hepatic steatosis with or without coexisting significant fibrosis. P-values are tested by the chi-squared test.

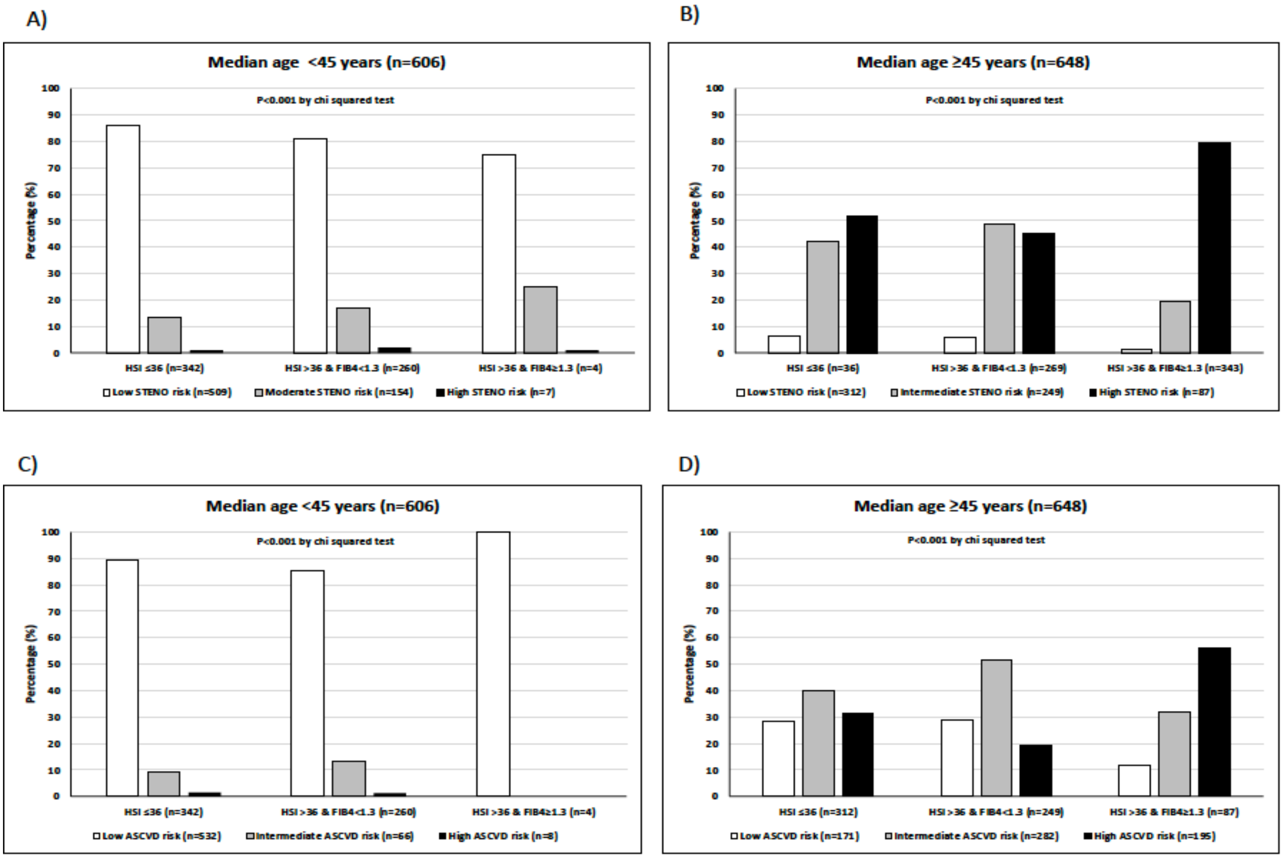

Supplementary Figure 5. Prevalence rates of the 10-year estimated CVD risk, using either the Steno type 1 risk engine (panels A and B) or the ASCVD risk calculator (panels C and D), in adult patients with T1DM who were simultaneously stratified by sex (men vs. women) and presence or absence of hepatic steatosis with or without coexisting significant fibrosis. P-values are tested by the chi-squared test.

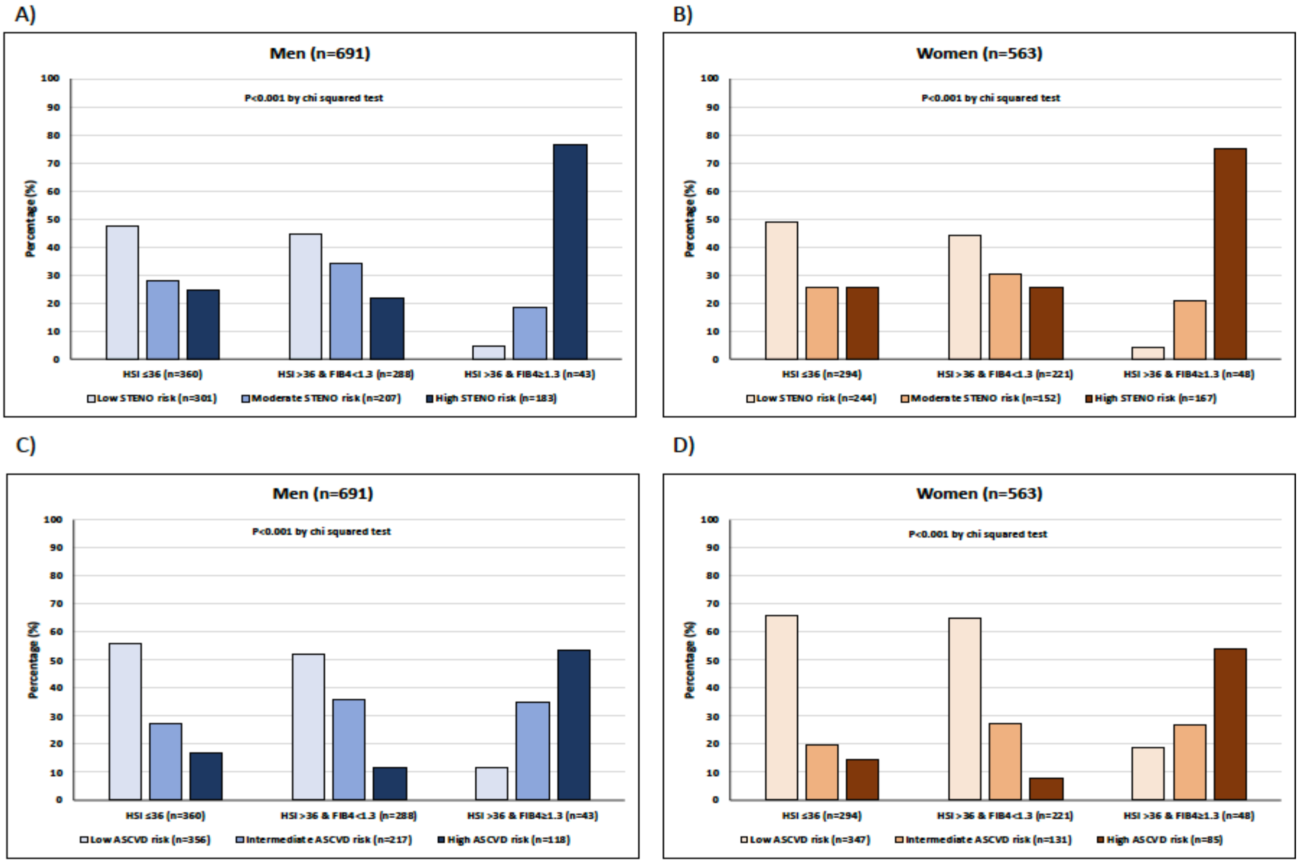

Supplementary Figure 6. Prevalence rates of the 10-year estimated CVD risk, using either the Steno type 1 risk engine (panels A and B) or the ASCVD risk calculator (panels C and D), in adult patients with T1DM who were simultaneously stratified by hypertension status (defined as blood pressure ≥140/90 or drug treatment) and presence or absence of hepatic steatosis with or without coexisting significant fibrosis. P-values are tested by the chi-squared test.

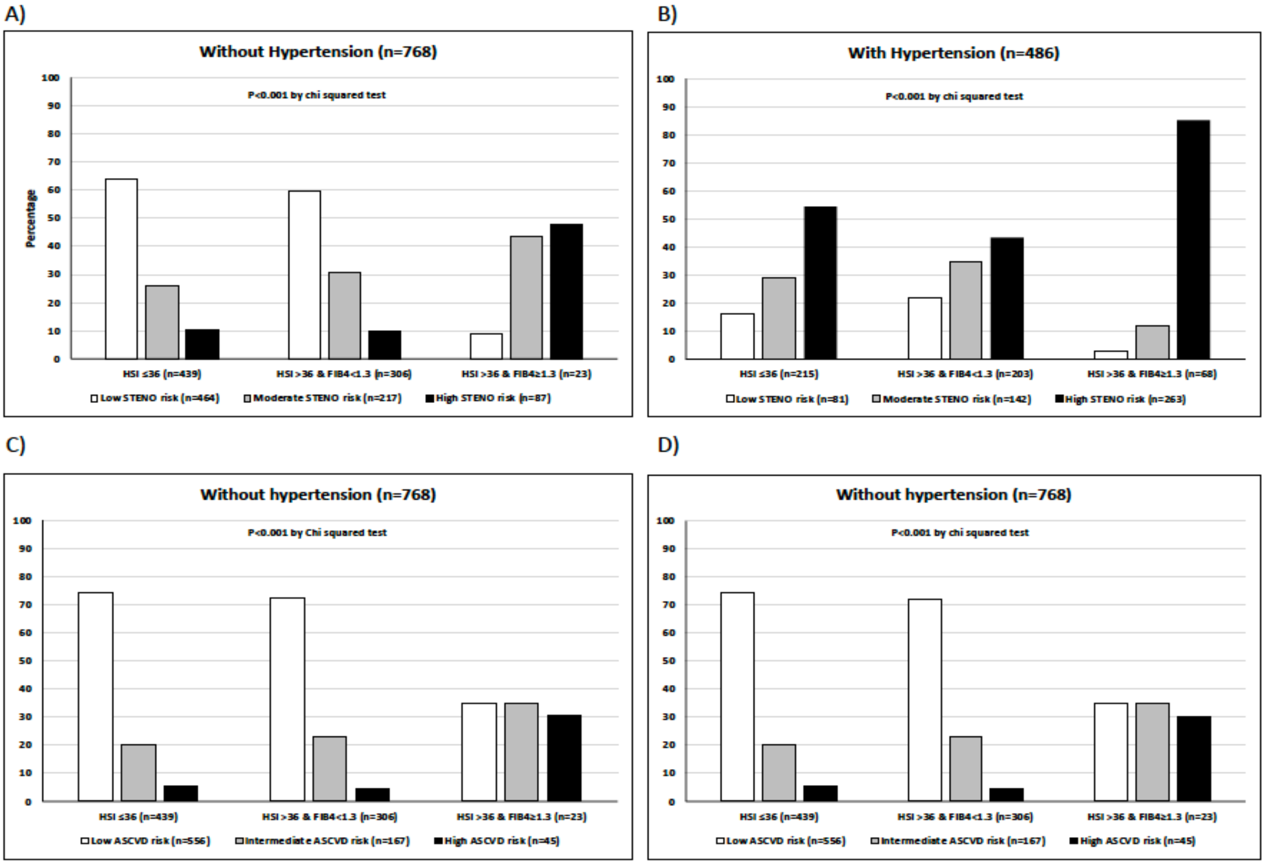

Supplementary Table 1. Association between hepatic steatosis with or without coexisting significant fibrosis and the 10-year estimated CVD risk (using either the Steno type 1 risk engine or the ASCVD risk score). In these logistic regression models, we excluded patients with intermediate HSI values (i.e., those with HSI values between 30 and 36) from the analysis.

**Logistic Regression Analyses**	**Odds Ratios (95% confidence intervals)**	***P*-value**
**Y= High or moderate risk *vs.* low Steno type 1 risk score**		
Unadjusted model		
Patients with HSI ≤30 (n=115)	Ref.	-
Patients with HSI >36 and FIB4 <1.3 (n=509)	1.32 (0.98-1.97)	0.180
Patients with HSI >36 and FIB4 ≥1.3 (n=91)	22.9 (7.88-66.6)	<0.001
Adjusted model 1		
Patients with HSI ≤30 (n=115)	Ref.	-
Patients with HSI >36 and FIB4 <1.3 (n=509)	0.96 (0.51-1.80)	0.897
Patients with HSI >36 and FIB4 ≥1.3 (n=91)	11.6 (3.22-41.9)	<0.001
Adjusted model 2 (n=600)		
Patients with HSI ≤36 (n=89)	Ref.	-
Patients with HSI >36 and FIB4 <1.3 (n=432)	0.81 (0.40-1.65)	0.568
Patients with HSI >36 and FIB4 ≥1.3 (n=79)	11.4 (2.74-47.9)	0.001
**Y= High or intermediate risk *vs.* low ASCVD risk score**		
Unadjusted model		
Patients with HSI ≤30 (n=115)	Ref.	-
Patients with HSI >36 and FIB4 <1.3 (n=509)	0.77 (0.51-1.16)	0.108
Patients with HSI >36 and FIB4 ≥1.3 (n=91)	5.79 (2.94-11.4)	<0.001
Adjusted model 1		
Patients with HSI ≤30 (n=115)	Ref.	-
Patients with HSI >36 and FIB4 <1.3 (n=509)	0.56 (0.31-1.02)	0.054
Patients with HSI >36 and FIB4 ≥1.3 (n=91)	2.64 (1.12-6.21)	0.026
Adjusted model 2 (n=1,043)		
Patients with HSI ≤36 (n=89)	Ref.	-
Patients with HSI >36 and FIB4 <1.3 (n=432)	0.47 (0.34-1.06)	0.056
Patients with HSI >36 and FIB4 ≥1.3 (n=79)	3.39 (1.27-9.12)	0.015

Cohort size, n=715, except where indicated. Data are expressed as odds ratio (OR) and 95% confidence interval, assessed by univariable and multivariable logistic regression analyses. The dependent variable of logistic regression models was: (a) the high or moderate Steno type 1 risk groups combined vs. the low Steno type 1 risk group, or (b) the high or Intermediate ASCVD risk groups combined vs. the low ASCVD risk group. Regression model 1 was adjusted for sex, BMI, diabetes duration, HbA1c, chronic kidney disease (defined as e-GFR <60 mL/min/1.73 m^2 or abnormal albuminuria), and lipid-lowering medication use. Regression model 2 was adjusted for the same model's 1 covariates after excluding those with significant alcohol intake (n=115).

Supplementary Table 2. Association between hepatic steatosis with or without coexisting significant fibrosis and the 10-year estimated CVD risk (using either the Steno type 1 risk engine or the ASCVD risk score). In these logistic regression models, the presence of NAFLD was defined as HSI >30 (instead of HIS >36 as reported in Table 4).

**Logistic Regression Analyses**	**Odds Ratios (95% confidence intervals)**	***P*-value**
**Y= High or moderate risk *vs.* low Steno type 1 risk score**		
Unadjusted model		
Patients with HSI ≤30 (n=115)	Ref.	-
Patients with HSI >30 and FIB4 <1.3 (n=918)	0.99 (0.67-1.46)	0.964
Patients with HSI >30 and FIB4 ≥1.3 (n=221)	16.9 (8.63-32.9)	<0.001
Adjusted model 1		
Patients with HSI ≤30 (n=115)	Ref.	-
Patients with HSI >30 and FIB4 <1.3 (n=918)	0.60 (0.34-1.02)	0.056
Patients with HSI >30 and FIB4 ≥1.3 (n=221)	11.6 (3.22-41.9)	<0.001
Adjusted model 2 (n=1,043)		
Patients with HSI ≤30 (n=89)	Ref.	-
Patients with HSI >30 and FIB4 <1.3 (n=768)	0.57 (0.31-1.05)	0.059
Patients with HSI >30 and FIB4 ≥1.3 (n=186)	9.88 (4.04-24.2)	<0.001
**Y= High or intermediate risk *vs.* low ASCVD risk score**		
Unadjusted model		
Patients with HSI ≤30 (n=115)	Ref.	-
Patients with HSI >30 and FIB4 <1.3 (n=918)	0.54 (0.37-1.01)	0.053
Patients with HSI >30 and FIB4 ≥1.3 (n=221)	4.92 (2.97-8.11)	<0.001
Adjusted model 1		
Patients with HSI ≤30 (n=115)	Ref.	-
Patients with HSI >30 and FIB4 <1.3 (n=918)	0.41 (0.20-1.01)	0.052
Patients with HSI >30 and FIB4 ≥1.3 (n=221)	2.05 (1.14-3.71)	0.017
Adjusted model 2 (n=1,043)		
Patients with HSI ≤30 (n=89)	Ref.	-
Patients with HSI >30 and FIB4 <1.3 (n=768)	0.35 (0.34-1.02)	0.056
Patients with HSI >30 and FIB4 ≥1.3 (n=186)	2.00 (1.03-3.89)	0.045

Cohort size, n=1,254, except where indicated. Data are expressed as odds ratio (OR) and 95% confidence interval, assessed by univariable and multivariable logistic regression analyses. The dependent variable of logistic regression models was: (a) the high or moderate Steno type 1 risk groups combined vs. the low Steno type 1 risk group, or (b) the high or Intermediate ASCVD risk groups combined vs. the low ASCVD risk group. Regression model 1 was adjusted for sex, BMI, diabetes duration, HbA1c, chronic kidney disease (defined as e-GFR <60 mL/min/1.73 m^2 or abnormal albuminuria), and lipid-lowering medication use. Regression model 2 was adjusted for the same model's 1 covariates after excluding those with significant alcohol intake (n=215).
